# Dystrophin (*DMD*) Missense Variant in Cats with Becker-Type Muscular Dystrophy

**DOI:** 10.3390/ijms24043192

**Published:** 2023-02-06

**Authors:** Stephanie Hilton, Matthias Christen, Thomas Bilzer, Vidhya Jagannathan, Tosso Leeb, Urs Giger

**Affiliations:** 1 Tierärztliche Klinik für Kleintiere, Robert-Bosch-Str. 8a, 61267 Neu-Anspach, Germany; 2 Institute of Genetics, Vetsuisse Faculty, University of Bern, Bremgartenstrasse 109a, 3001 Bern, Switzerland; 3 Institut für Pathologie Leverkusen (E. Kilic), Paracelsusstrasse 17, 51375 Leverkusen, Germany; 4 Vetsuisse Faculty, University of Zürich, Winterthurerstrasse 260, 8057 Zürich, Switzerland

**Keywords:** *Felis catus*, feline, myopathy, creatine kinase, Duchenne, mutation, hereditary disease, X-linked, precision medicine

## Abstract

Muscular dystrophy due to dystrophin deficiency in humans is phenotypically divided into a severe Duchenne and milder Becker type. Dystrophin deficiency has also been described in a few animal species, and few *DMD* gene variants have been identified in animals. Here, we characterize the clinical, histopathological, and molecular genetic aspects of a family of Maine Coon crossbred cats with clinically mild and slowly progressive muscular dystrophy. Two young adult male littermate cats exhibited abnormal gait and muscular hypertrophy with macroglossia. Serum creatine kinase activities were highly increased. Histopathologically, dystrophic skeletal muscle exhibited marked structural changes including atrophic, hypertrophic, and necrotic muscle fibers. Immunohistochemistry showed irregularly reduced expression of dystrophin but the staining of other muscle proteins such as β- and γ-sarcoglycans as well as desmin was also diminished. Whole genome sequencing of one affected cat and genotyping of the littermate found both to be hemizygous mutant at a single *DMD* missense variant (c.4186C>T). No other protein-changing variants in candidate genes for muscular dystrophy were detected. In addition, one clinically healthy male littermate was hemizygous wildtype, while the queen and one female littermate were clinically healthy, but heterozygous. The predicted amino acid exchange (p.His1396Tyr) resides in a conserved central rod spectrin domain of dystrophin. Various protein modeling programs did not predict major disruption of the dystrophin protein by this substitution, but the altered charge of the region may still affect protein function. This study represents the first genotype-to-phenotype correlation of Becker-type dystrophin deficiency in companion animals.

## 1. Introduction

Dystrophin deficiency was discovered in 1986 as the major cause of X-linked recessive muscular dystrophy in humans and thereafter also described in several domestic animal species [1,2,3,4]. Dystrophin-deficient dogs exhibit very similar clinical features, and thus became the favorite animal model to investigate this disorder and assess novel therapies at the preclinical stage (Online Mendelian Inheritance in Animals [OMIA] #001081-9615) [5,6,7]. In contrast to dystrophin-deficient humans and dogs, cats with X-linked muscular dystrophy exhibit predominantly muscular hypertrophy rather than dystrophy (OMIA #001081-9685) [8,9,10,11,12,13,14,15]. More recently, several juvenile pigs with dystrophin deficiency or insufficiency have been described (OMIA #001081-9823 [Duchenne]; #001888-9823 [Becker]) and few became useful disease models [16,17,18,19,20,21,22,23]. However, naturally occurring and genetically engineered models in mice [24,25,26,27], rats [28], rabbits [29], and pigs [16,17,20,22] did not or only selectively show the classic muscular dystrophy and atrophy as observed in human and canine patients. 

Based on disease onset, progression, and clinical phenotype, dystrophin deficiency in humans is differentiated between a severe Duchenne- and milder Becker-type muscular dystrophy [30,31]. Moreover, the discovery of likely pathogenic *DMD* variants revealed that the clinical features of X-linked muscular dystrophy correlate well with the *DMD* genotype in humans. Human patients with Duchenne muscular dystrophy have typically nonsense or frameshift *DMD* variants with complete loss of dystrophin protein function. In contrast, those with Becker type have missense and other milder *DMD* variants allowing for varied dystrophin expression and function [32,33,34,35]. 

Domestic animals with dystrophin deficiency mostly exhibit a severe phenotype with the juvenile onset of, and the rapidly progressive myopathy which corresponds to, the Duchenne-type muscular dystrophy [3,4,17,25]. Only a few clinicopathological reports in domestic animals suggest the presence of a milder disease course in dogs and pigs and thus potentially reflect Becker-type muscular dystrophy [18,23,36,37,38]. We report here on adult male domestic cats with a clinically mild-to-moderate and slowly progressive dystrophic myopathy course. Histopathology, histochemistry, and immunohistochemistry revealed moderate-to-severe structural changes and irregular expression of dystrophin and other proteins, and a missense *DMD* variant likely causing X-linked recessive muscular dystrophy, which mimics Becker-type muscular dystrophy in human patients.

## 2. Results

### 2.1. Clinical Manifestations

A 2½-year-old castrated male domestic cat (index case, cat #1) and its male littermate (cat #2) were presented to the Tierärztliche Klinik für Kleintiere, Neu-Anspach, Germany, because of clumsy gait, difficulty jumping and grooming, and protrusion of the tongue tip (video in supplementary materials). These cats lived in- and outdoors in a suburban neighborhood without any major physical impediments according to their owners. No information of prior physical examinations, medical histories, and laboratory test results was available.

**Figure 1:**
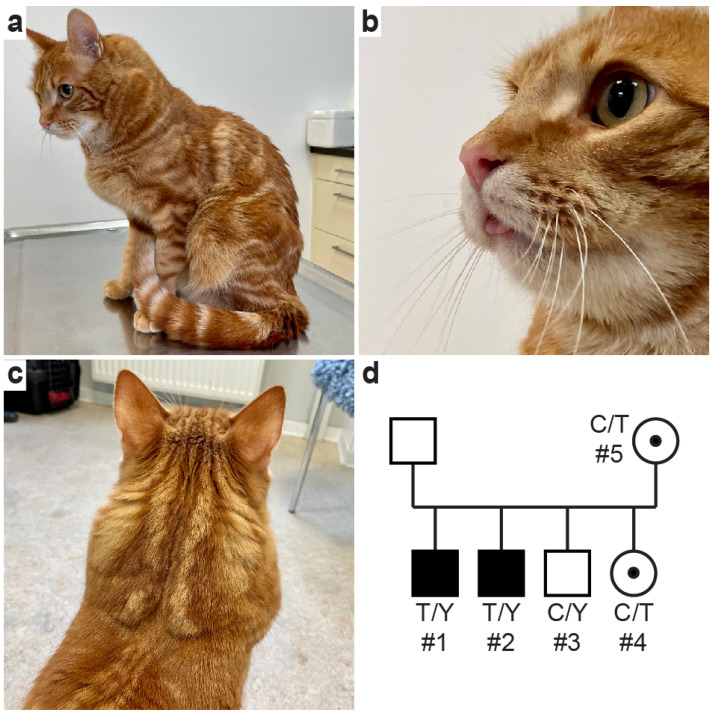
(**a**–**c**) Clinical images of affected cat #1, with muscle hypertrophy and protrusion of the tongue tip; (video in supplementary materials), (**d**) Pedigree of Maine Coon crossbred domestic cat family with muscular dystrophy. The affected males are depicted as black squares; clinically unaffected males are shown as empty symbols. The unaffected female littermate cat #4 and queen cat #5 were heterozygous for the mutant allele (C/T); symbols with black dots indicate carrier status for an X-linked trait. Genotypes for the *DMD*:c.4186C>T variant are indicated; the “Y” indicates the hemizygous genotype in males with either a mutant or wildtype X-chromosomal allele. The queen’s mother was a Maine Coon cat but not available for study. The litter’s tom was also not available for clinical examination and genotyping.

Physical examinations of the two affected male littermates revealed a normal body condition score but marked generalized muscular hypertrophy, particularly of the neck and upper limbs, as well as macroglossia and a more forceful breathing pattern (Figure 1). There was no muscle cramping and dimpling, making a congenital myopathy unlikely. Imaging of both the body cavities and heart of cat #1 and cat #2 did not reveal any abnormalities besides the systemic skeletal muscular hypertrophy. There were no cardiac murmurs auscultated, pulse rate and quality appeared normal, and echocardiogram parameters were in normal reference intervals (Figure S1), thus providing no clinical evidence of a cardiomyopathy. Furthermore, there was neither clinical nor radiographic evidence of megaesophagus in either of the affected cats.

While the tom of this litter remains unknown, one male (cat #3) and one female littermate (cat #4) and the queen (cat #5) of the affected litter—all living in the same neighborhood—showed neither muscular hypertrophy nor other clinical abnormalities on physical examination. According to the owner, the queen was a Maine Coon x domestic shorthair crossbred cat, and the breed of the tom remained unknown. Compared to the healthy appearing littermates, the two affected males appeared to have longer hair coats, bushy tails, and ear tufts suggesting Maine Coon breed ancestry. The results of a commercial feline DNA panel of cat #1 revealed a 26% Maine Coon contribution, a blood type *A* by *CMAH* genotyping, and no known disease-causing variants tested for (Mars Veterinary, Portland, OR, USA) [39,40]. The conditions of both affected males only marginally progressed clinically, and the two healthy appearing littermates as well as the litter’s queen also remained unchanged over the eight-month observation period.

### 2.2. Blood Test Results

The serum creatine kinase activities of the two male affected cats were extremely high at initial examination as well as after eight months compared to the other male and female littermate, queen, and reference intervals (Table 1). In addition, the serum alanine (ALT) and aspartate aminotransferase (AST) activities were moderately increased in both affected males consistent with a dystrophic myopathy. All other routine blood parameters including complete blood cell count and serum chemistry results of both affected males were within reference intervals. Moreover, serum creatine kinase activities of the male and female littermates and queen of the litter were within the reference interval (Table 1).

### 2.3. Histopathology and Immunohistochemistry

**Figure 2:**
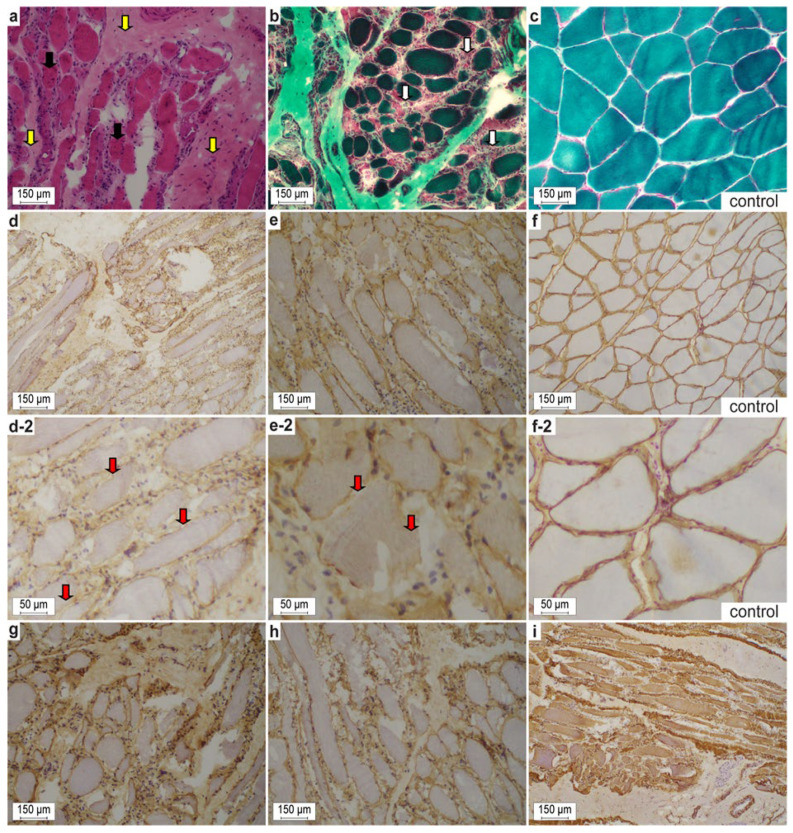
(**a**,**b**,**d**,**e**,**g**–**i**) Representative cryosections of the affected gastrocnemius muscle from the dystrophic Maine Coon crossbred domestic cat #1. (**a**,**b**) Noteworthy are the variability of myofiber sizes, degenerative (groups of necrotic fibers with myophagocytosis, white arrows) and regenerative muscle features (small fibers with prominent vesicular central nuclei; multiple nuclear internalization, black arrows), and endomysial fibrosis (yellow arrows) in affected cat #1. (**d**,**e**) On immunohistochemistry, membranous dystrophin often is discontinuous or even absent in between individual fibers (red arrows). Moreover, γ- and β-sarcoglycans (**g**,**h**) and desmin (**i**) were diminished and interrupted. (**c**,**f**) control sections from a European domestic shorthair cat. (**a**) H&E stain. (**b**,**c**) Gomori trichrome stain according to Engel. (**d**,**f**) Dystrophin 1 (1:100, DYS1). (**e**) Dystrophin 2 (1:100, DYS2) at regular and increased magnification. (**g**) γ-sarcoglycan (1:100, γ-SARC). (**h**) β-sarcoglycan (1:50, β-SARC). (**i**) desmin (1:50, Desmin Clone D33 M0760). (**c**,**f**) Control sections of a domestic short hair cat.

Transverse and longitudinal sections of gastrocnemius muscle obtained from cat #1 showed structural changes consistent with a dystrophic myopathy (Figure 2): Bimodal pathological fiber size variations, comprising multiple enlarged and atrophic myocytes, as well as muscle fiber necrosis, were present. Furthermore, chronic diffuse myofibrosis, nuclear internalization, and interstitial lymphocytic infiltration were seen. No ragged-red myofibers, cones, rods, cores, and targets were seen, but there were myocytes with increased fibrils and clumping of the myotubular apparatus. Very mild hypomyelination was noted in endomysial nerve fibers (Figure 2a,b). Enzyme histochemistry and myosin heavy chain immunohistochemistry revealed normal type 1 and 2 fiber distribution. Mild increases in subsarcolemmal and interfibrillar lipid droplets were found in a few muscle fibers. Positive acid phosphatase activity highlighted necrotizing myofibers. Together, these histopathological features were consistent with a dystrophic myopathy as previously described in domestic cats with X-linked muscular dystrophy [8,9,10,11,12,13,14,41]

**Figure 3:**
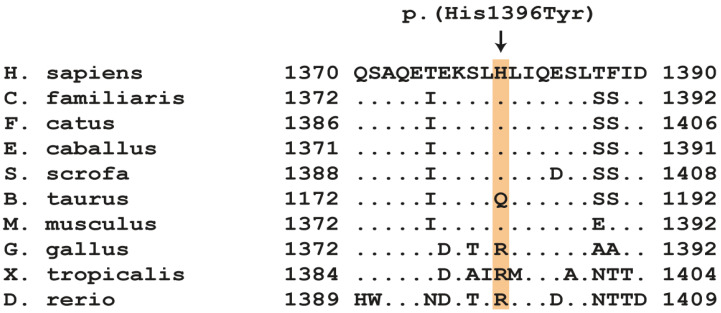
Amino acid alignment of a part of dystrophin’s spectrin 10 domain in several species surrounding the p.(His1396Tyr) variant. The *DMD* variant affects a histidine residue, which is mostly conserved among mammals. Accession numbers: human (*H. sapiens*) NP_003997.1; dog (*C. familiaris*) NP_001003343.1; cat (*F. catus*) XP_044906722.1; horse (*E. caballus*) XP_023489578.1; domestic pig (*S. scrofa*) XP_020935202.1; domestic cattle (*B. taurus*) XP_002700291.2.; mouse (*M. musculus*) NP_031894.1; chicken (*G. gallus*) NP_990630.1; western clawed frog (*X. tropicalis*) XP_031752861.1; zebrafish (*D. rerio*) NP_001313616.1.

Immunohistochemistry of muscle from cat #1 revealed irregular and partially deficient expression of both DYS1 (rod domain) and DYS2 (carboxy terminus) (Figure 2d,e,d-2,e-2). Moreover, antibody reactivity of β- and γ-sarcoglycans (Figure 2g,h) as well as β-dystroglycan was diminished and uneven compared to the feline controls. Furthermore, the muscular intermediate filament desmin was severely interrupted (Figure 3). Despite the severe structural muscle fiber disturbances, the dystrophin remains identifiable in the dystrophin-associated muscle protein complex. The antibody reactions against DYS3 (amino terminus), ß-dystroglycan, δ-sarcoglycan, and also α2-laminin were weak or negative against the control as well as cat #1 muscle proteins, thus precluding any interpretation.

### 2.4. Molecular Genetics

The whole genome of dystrophic cat #1 was sequenced and searched for private homozygous and hemizygous variants that were not present in the genome sequences of 74 control cats (Table 2 and Table S2).

Analysis of the detected variants revealed only 18 private protein-changing variants in 13 genes (Table S3). Seventeen did not affect known musculoskeletal genes and thus were unlikely responsible for the myopathy in the index case. One single nucleotide variant (SNV) was in the *DMD* gene representing a prime candidate gene for a hereditary dystrophic myopathy. This *DMD* missense variant in exon 30 constitutes a G>A exchange at position 27,988,938 on the short arm of the X-chromosome, XM_045050787.1:c.4186C>T. This variant is predicted to result in a histidine-to-tyrosine substitution in the DMD protein, XP_044906722.1:p.(His1396Tyr). The predicted amino acid substitution lies within the spectrin 10 domain of the protein, which is conserved among mammals (Figure 3). The His1396Tyr substitution was not predicted to be pathogenic by several in silico prediction tools (Table 3). A homologous amino acid exchange, NP_003997.2:His1380Tyr, was observed once in the *DMD* gene of a hemizygous adult male person according to the gnomAD database but without further clinicopathological information. The gnomAD ID for the variant is “X-32429964-G-A” [42].

**Figure 4:**
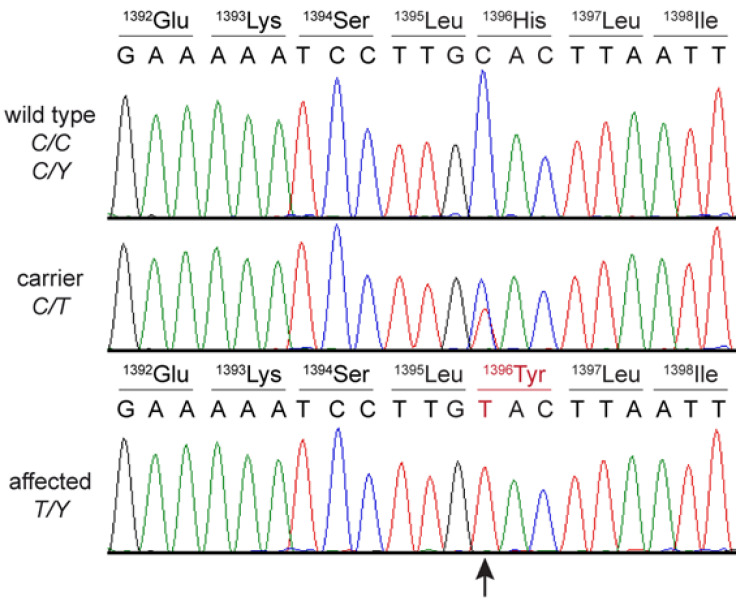
Sanger sequencing electropherograms of a wildtype, female carrier cat #4, and dystrophic male cat #2 surrounding the *DMD*:c.4186C>T variant. Amino acid changes in carrier female and the dystrophic cat #2 are shown.

The presence of the *DMD* missense variant was confirmed by Sanger sequencing in both dystrophic male cats (Figure 4). The unaffected male littermate was hemizygous wildtype, while the female littermate and queen were heterozygous for the mutant allele (Figure 1d). The genotype–phenotype correlation was consistent for an X-linked recessive mode of inheritance. The variant was additionally genotyped in 277 control Main Coon cats and 320 additional control cats of diverse other breeds with similar sex distribution. None of these cats carried the mutant allele.

## 3. Discussion

Duchenne-type muscular dystrophy, characterized by an early severe disease course and complete dystrophin deficiency caused by *DMD* null variants, has been previously reported in different breeds of domestic dogs (OMIA #001081-9615) [5,6,46,47,48,49,50], domestic shorthair as well as Maine Coon cats (OMIA #001081-9685) [2,3,4,8,9,11,41,51], pigs [16,17,19,20], mice [25,26], rats [28], and rabbits [29]. In contrast, Becker-type muscular dystrophy has been rarely reported in domestic animals, e.g., in dogs [36,37,38] and pigs (OMIA #001888-9823) [18,23]. The here-reported two male Maine Coon crossbred cats with an adult onset and slowly progressive disease course, histopathologically a dystrophic myopathy with diminished but positive dystrophin staining and thus positive cross reacting material (CRM+), and a single *DMD* missense variant, support a diagnosis of Becker-type muscular dystrophy.

Based upon the whole genome sequencing of one of the two dystrophic cats, with a specific focus on musculoskeletal genes, the only private protein-changing variant in a known functional candidate gene was identified in the *DMD* gene. The single nucleotide exchange (c.4186C>T) is predicted to lead to an amino acid substitution (His1396Tyr), which resides in the center rod spectrin 10 domain—a conserved domain among all mammals except cattle (Figure 3). The amino acid substitution is predicted to remove a single positive charge, but protein structure and function may not be seriously affected based upon the various functional prediction programs applied. According to the gnomAD variant database [42], a missense variant leading to an exchange from histidine to tyrosine at the homologous position in the human DMD protein (NP_003997.2:His1380Tyr) has been once observed in an older man, but there are no descriptions whether or not this person had a dystrophic phenotype. Many *DMD* variants associated with Becker-type muscular dystrophy are missense variants and show only minor impacts with protein modeling on dystrophin protein structure and function [32,33,52]. No protein function studies were performed to prove causality of the missense variant of dystrophic cats described here in vitro or in vivo.

When genotyping the available family members for the *DMD*:c.4186C>T SNV, the other affected male was also hemizygous for the mutant allele, the queen and a female littermate were heterozygous and a male littermate was hemizygous wildtype at the SNV. The expected co-segregation of genotypes with the observed dystrophy was consistent with an X-linked recessive mode of inheritance. The tom of the litter and mother of the queen were not available for genotyping. The queen as well as affected males had features of the Maine Coon breed, and indeed cat #1 had 26% SNV alleles seen in Maine Coon cats [39]. The other littermates appeared more like regular domestic shorthair cats. Interestingly, a *DMD* nonsense variant causing Duchenne-type muscular dystrophy in a Maine Coon family was just reported [51].

The identification of a likely candidate causal variant would allow genetic testing to reliably identify additional carrier females. However, the mutant *DMD* allele was absent from 597 unrelated control cats mostly sampled in Switzerland, including 277 Maine Coon cats. It is unclear how widely the mutant *DMD* allele may have spread in the regional cat population in Germany, but it is not anticipated that this *DMD* missense variant is widespread.

Consistent with an amino acid substitution in dystrophin, which is not predicted to drastically change the protein structure and stability, immunohistochemistry for dystrophin protein was CRM+. All previously reported cats with dystrophin deficiency were CRM- including the recently reported two Maine Coon cats with Duchenne muscular dystrophy [51]. Accurate quantification would have been helpful but could not be performed because no samples were available. Dogs with muscular dystrophy show muscle dystrophy and atrophy with sometimes macroglossia [4], and various deleterious *DMD* variants have been described in several canine breeds (OMIA #001081-9615) [6,46,47,49]. While in most affected dogs, dystrophin is absent by immunohistology, few cases have been described having a partially truncated dystrophin and a slower disease progression [36,37,38]. For instance, there was one nine-year-old male Border terrier where the immunohistochemical staining of the amino-terminal dystrophin protein was present but the carboxy terminal was patchy and reduced, suggesting an 80 kDa truncated protein. However, no molecular genetic studies were pursued to define the *DMD* variant [38].

The staining for dystrophin and other musculoskeletal proteins was noticeably reduced in the affected cat studied here. None of the musculoskeletal proteins appeared completely deficient in the affected cat #1 of this report and no protein altering DNA variants were found in any of the corresponding genes except *DMD*. It is well recognized that dystrophin deficiency and dysfunction are associated with impaired stability of the entire dystrophin-associated protein complex, and thus reduced amounts of any of its muscle proteins may be expected [53]. Furthermore, previously studied dystrophin-deficient cats also exhibited reduced complex proteins [12]. All previously reported cats were classified as having Duchenne-type muscular dystrophy, including the recently described dystrophic Maine Coon cat [51]. Likewise, human patients with Duchenne-type as well as Becker-type muscular dystrophy have varying degrees of reduced muscle dystrophin-associated complex proteins [54,55].

Extremely high serum CK activities are a hallmark finding in all dystrophin-deficient humans and domestic animals. The dystrophic cats reported here had high serum CK activity, elevated 30–70× compared to the upper limit of normal reference intervals as previously described with other dystrophic cats [8,9,13,41] (except one reported cat with an unexplained normal serum CK activity [14]). In contrast, human patients and animals including cats with laminin and other dystrophin-associated complex protein deficiencies have only mildly to moderately increased serum CK activities [56,57,58]. The increase in serum CK activities does not appear to allow differentiation between Duchenne and Becker type in cats particularly as cats may massively increase their serum CK activity with anesthesia and stress.

A unique feature of all thus-far reported dystrophin-deficient cats, including the two males of this study, is that on clinical examinations they phenotypically exhibit predominantly muscle hypertrophy rather than muscle atrophy [3,8,9,13,14,15,41]. In that vein, the phenotype of a dystrophic cat may be confused with disorders such as myotonia congenita. However, those also exhibit muscle cramps and dimpling and have only mildly increased CK values [59]. The appendicular muscle hypertrophy and macroglossia were recognized at juvenile age in all previously described cats but were not noted until adulthood in the dystrophic cats reported here. The later onset is consistent with the adult onset of human patients with Becker-type muscular dystrophy [31]. Becker-type dystrophy has an approximate onset of 30 years in human patients and progressing slowly, while Duchenne type is typically evident in boys in early childhood and progressing rapidly [31]. Over the eight-month observation period, only mild clinical progression was noted in the two young adult dystrophic cats reported here.

There are various potential genetic and environmental modifiers affecting the clinical manifestations of dystrophin deficiency in humans and animals [60,61]. Interestingly, the cytidine monophospho-N-acetylneuraminic acid hydroxylase (CMAH) activity has been associated with protecting against severe clinical signs of dystrophin deficiency. To that end, *DMD* and *CMAH* double knockout mice exhibit muscular dystrophy, while those with only dystrophin deficiency remain clinically mostly unaffected [61]. In humans, the *CMAH* gene contains a large deletion and thus is a dysfunctional pseudogene, which may in part explain the more severe clinical manifestations in dystrophin deficient human patients [62]. In contrast, in domestic cats CMAH activity is determining the clinically important *AB(C)* blood group [40]. Most cats are type *A* and have a functional CMAH enzyme converting an N-acetyl into a N-glycolyl-neuraminic acid group. The two dystrophic cats have blood type *A* by phenotypic evaluation and did not harbor any of the four variants screened for in purebred cats to cause blood type *B* and *AB(C)* [40]. The blood types of the other described dystrophic cats in the literature were not reported but were also likely having type *A* blood as it is the most common blood type in domestic cats [40]. It remains unknown if the phenotype of the dystrophic cats could be more severe with non-functional *CMAH* and blood type *B*.

Dystrophin deficiency also affects cardiac muscles and leads to dystrophic cardiomyopathy in dystrophic human patients [63] and large animal models such as dogs [64], pigs [16], and cats [65]. No cardiac murmurs were noted in either of the dystrophic cats in this report and cardiac imaging studies did not indicate any cardiac abnormalities even at 3.5 years of age. However, these cats may still develop a cardiomyopathy as they age. Likewise, the two dystrophic cats in this report had no clinical and imaging evidence of megaesophagus reported in other dystrophic cats of the Duchenne type [3].

Among the dystrophic animal models, the dystrophic golden retrievers were the first model that phenotypically most closely mimics the Duchenne muscular dystrophy in human patients. This animal-disease model has been extensively used to study pathogenesis as well as to evaluate the safety and efficacy of novel therapies over the past three decades [5,66].

Pigs are the only other domestic species in which their naturally occurring muscular dystrophy has been well characterized [18]. Recently, a 6-month-old pig with macroglossia at a slaughterhouse was found to have severely reduced dystrophin staining and a pseudoexon insertion was reported. While those authors suggested this to be a Becker-type dystrophy, the early onset, near complete lack of dystrophin staining and *DMD* insertion predicting a CRM- pathology makes this more likely a Duchenne-type dystrophin deficiency [23]. Interestingly, a couple of decades ago, young piglets thought to have porcine stress syndrome at slaughterhouses had reduced dystrophin protein and *DMD* missense mutations, thereby resembling Becker-type dystrophin deficiency [67]. Finally, porcine models of Duchenne muscular dystrophy have been engineered by targeted deletion of *DMD* exon 52 in porcine kidney cells followed by somatic cell nuclear transfer to generate affected male *DMD*^Y/-^ piglets, in the first run, and breeding with newly generated female *DMD*^+/-^ carriers on the second. *DMD*^Y/-^ piglets appear to be a close homologue for severe Duchenne muscular dystrophy in humans based upon clinical, biochemical, and pathological examination, including cardiomyopathy [16,17,19,20,22]. The majority of dystrophin-deficient piglets die within the first weeks of life, which might be extended with intense neonatal care to a few months of age. Since there is only one reproducible large animal model available yet for Becker-type dystrophy [18,19], clinical cases of subtotal dystrophin deficiency in companion animals may narrow the gap offering additional insights into the pathobiology of Becker-type dystrophin deficiency.

When applying the guidelines for the interpretation of DNA sequence variants in human genetics [68] to the herein investigated cats, four arguments lead to our classification of the *DMD*:c.4186C>T variant. One pathogenic strong (PS3), one pathogenic moderate to supporting (PM2), as well as two pathogenic supporting (PP1 and PP4) criteria taken together are enough to classify the sequence variant as “likely pathogenic” [68].

## 4. Materials and Methods

### 4.1. Animals and Samples

A family of random-bred domestic cats with partial Maine Coon ancestry, two of which were adult male cats with muscular hypertrophy, were clinicopathologically and genetically investigated. The clinical features, blood test results, and pathological examinations were performed as part of the routine diagnostic approach and for clinical management. The molecular genetic studies with DNA samples were part of an approved research study (the Cantonal Committee for Animal Experiments Bern; permit BE 71/19). Routine physical and cardiac examinations, hematology and serum chemistry tests, as well genetic testing for breed, hereditary disease, and other genetic traits (e.g, *ABC* blood typing) (Mars Veterinary, Portland, OR, USA) [39], were performed.

A biopsy from gastrocnemius muscle (~10 × 8 × 3 mm) was surgically obtained from one affected male cat (index case cat #1) at 2½ years of age and sent to the neuromuscular referral laboratory (Institute for Pathology, Klinikum Leverkusen, Germany) for diagnostic examination.

Ethylenediaminetetraacetate (EDTA)-anticoagulated blood samples for genetic analyses were obtained from index case cat #1 and were also available for family members (cat #2-5). In addition, archived genomic DNA samples from 597 domestic cats without evidence of muscular dystrophy of different breeds from the Vetsuisse Biobank were screened for the herein discovered *DMD* missense variant. Cheek swabs were used for commercial feline genetic marker screening (Mars Veterinary, Portland, OR, USA) [39].

### 4.2. Histological and Immunohistological Skeletal Muscle Investigations

Histopathological, histochemical, as well as immunohistochemical studies were performed as described [69,70,71]. Briefly, cryosections (10 μm) and paraffin-embedded sections (4–6 μm) of longitudinal and transverse skeletal muscle biopsies were stained with hematoxylin & eosin (H&E), Engel’s modified Gomori, Masson-Trichrome Elastica van Gieson (EvG), oil red O, periodic acid Schiff and/or Tibor Pap silver impregnation stains.

Antibodies for immunohistochemical stains of dystrophin 1 (1:100, DYS1), dystrophin 2 (1:100, DYS2), β-sarcoglycan (1:50, β-SARC), and γ-sarcoglycan (1:100, γ-SARC) from Novocastra Laboratory, Newcastle upon Tyne, UK, and desmin (clone D33M0760 from Agilent Dako, Santa Clara, CA, USA) were applied. All tissue sections were examined by a veterinary pathologist (T.B.) with brightfield microscopy.

### 4.3. Molecular Genetic Studies

Genomic DNA was isolated with standard protocols and the genome of cat #1 was sequenced at approximately 28.2× coverage with Illumina 2 × 150 bp paired end reads (Nova Seq 6000, Illumina Inc., San Diego, CA, USA). The data were analyzed as described previously [72] and submitted to the European Nucleotide Archive under sample accession no. SAMEA14502951.

Variant calling was performed using GATK Haplotype Caller [73] in gVCF mode [72]. Functional effects of called variants were predicted with the SnpEff software [74], together with the F. catus_Fca126_mat1.0 reference genome assembly and annotation release 105. For variant filtering, genomes from 74 control cats were included in the analysis (Table S2). PredictSNP [43], PROVEAN [44], and MutPred2 [45] were used to predict biological consequences of the discovered protein variant. Numbering in the feline *DMD* gene used here corresponds to the NCBI RefSeq accession numbers XM_045050787.1 (mRNA) and XP_044906722.1 (protein).

Available family members of the affected cat #1 and 597 other cats from the Vetsuisse Biobank with similar even sex distribution and without evidence of myopathy were genotyped, including 277 Maine Coon cats. Genomic DNA was amplified using AmpliTaq Gold 360 Master Mix (Thermo Fisher Scientific, Reinach, Switzerland). Primers 5′-TCC ATA CAA TGA GCC GCA TA-3′ (Primer F) and 5′-ACC GCG AGT AAG CAT TTC AC-3′ (Primer R) were used for generation of an amplicon containing the *DMD*:c.4186C>T variant. Direct Sanger sequencing of the PCR amplicons on an ABI 3730 DNA Analyzer (Thermo Fisher Scientific) was performed after treatment with exonuclease I and alkaline phosphatase (BioConcept Ltd., Allschwil, Switzerland). The Sanger sequences were analyzed using the Sequencher 5.1 software (Gene Codes, Ann Arbor, MI, USA).

## 5. Conclusions

In conclusion, based upon the likely X-linked recessive inheritance, the later and milder clinical muscle manifestations, the persistently high serum CK activities, the histopathological muscular dystrophy, and a single *DMD* missense variant, we believe this family represents the first clinical-to-molecular genetic characterization of a Becker type and thus cross-reactive material positive (CRM+) dystrophin deficiency in cats.

## Figures and Tables

**Table 1:** Select serum enzyme activities and *DMD* missense genotyping results in a family of Maine Coon crossbred domestic cats with X-linked muscular dystrophy.

Cat	Phenotype and Relationship	Age (Years)	Serum Enzyme Activities (IU/L)			*DMD*:c.4186C>T Genotype
			CK	AST	ALT	
#1	Index Case, Affected Male	2.5	15,597	364	364	T/Y
		3.2	23,085	477	369	
#2	Affected Male Littermate	2.7	30,939	608	365	T/Y
		3.2	38,814	732	472	
#3	Unaffected Male Littermate	2.5	136	ND	ND	C/Y
#4	Unaffected Female Littermate	2.5	177	ND	ND	C/T
#5	Unaffected Queen	3.5	235	ND	ND	C/T
Reference Interval		Adults	52–250	14–71	37–175	C/Y or C/C

Serum enzyme activities: CK, creatine kinase; AST, aspartate aminotransferase; ALT, alanine aminotransferase; ND, not determined; T/Y, hemizygous mutant; C/Y, hemizygous wildtype; C/T and C/C, heterozygous and homozygous wildtype females, respectively.

**Table 2:** Filtering for private protein-changing variants in dystrophic cat #1 against 74 control genomes. Only homozygous or hemizygous variants are reported (also Table S3).

Filtering Steps for Dystrophic Cat	Variants in Dystrophic Cat #1	
	Autosomes	X-Chromosome
All Variants	4,339,302	180,936
Protein-Changing Variants	35,548	305
Private Variants	1881	2367
Private Protein-Changing Variants	8	10
Private Variants in Muscle Genes	0	1

**Table 3:** Output of different in silico protein prediction tools for the feline DMD:p.(His1396Tyr) substitution.

Prediction Tool	Variant Score/Accuracy	Score for Deleterious Prediction
PredictSNP [43]	74% benign	n.a. *
PROVEAN [44]	−0.670	<−2.5 **
MutPred2 [45]	0.101	>0.8 ***

* PredictSNP takes output from different prediction tools and calls for either a benign or a deleterious prediction with an associated accuracy of the prediction. ** Specificity 80% and sensitivity 79% at this cutoff. *** False positive rate of 5% at this cutoff.

## Data Availability

The accessions for the sequence data reported in this study are listed in Table S2.

## Supplementary Materials

The following supporting information can be downloaded at: https://www.mdpi.com/article/10.3390/ijms24043192/s1.
