# Supplementary material for: Dystrophin (DMD) Missense Variant in Cats with Becker-Type Muscular Dystrophy
Source: Int J Mol Sci. 2023 Feb 6;24(4):3192. doi: 10.3390/ijms24043192 (PMC9964367; doi:10.3390/ijms24043192)
Supplement: Supplementary file 1 [file ijms-24-03192-s001.zip › S1_Cardio..pdf]

Supplement S1: Chest radiograph and echocardiographic images from cat #1 at 2 ½ years of age, illustrating normal cardiac size and function.

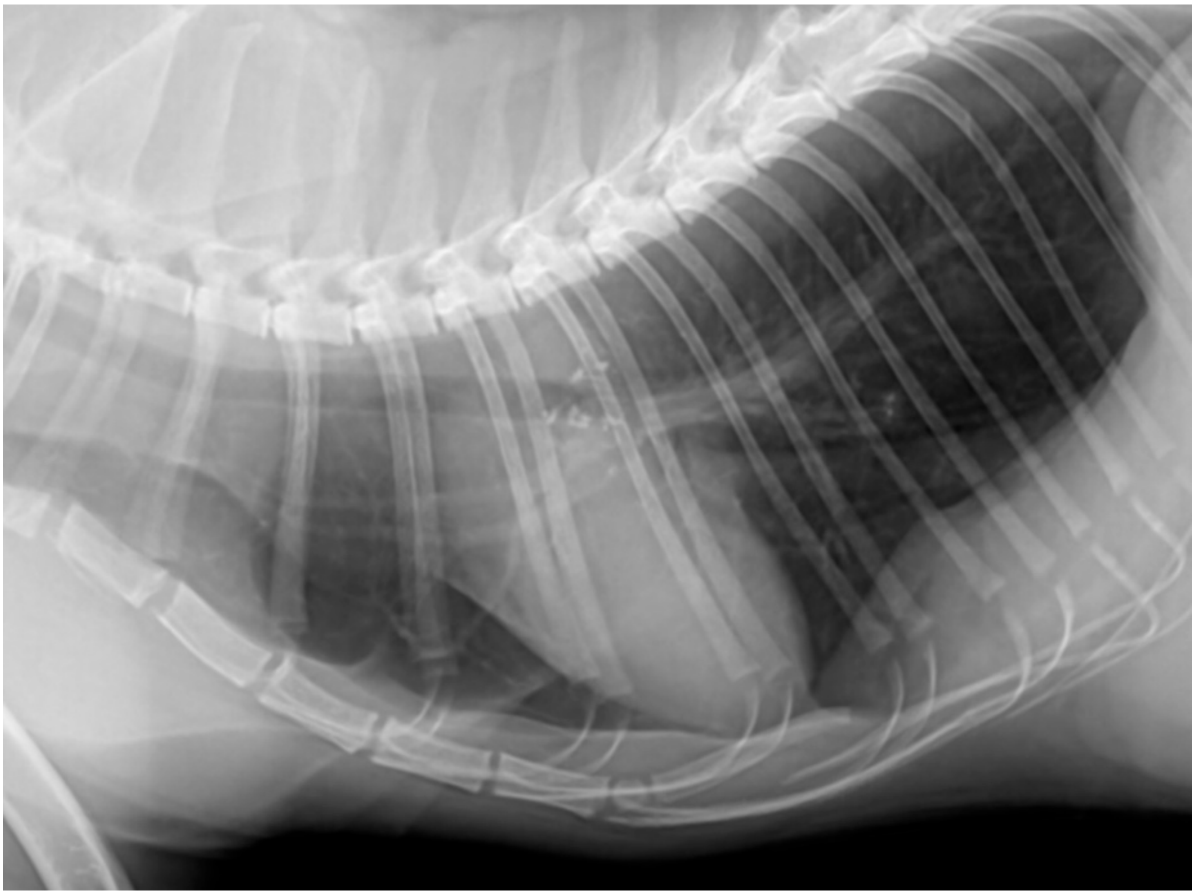

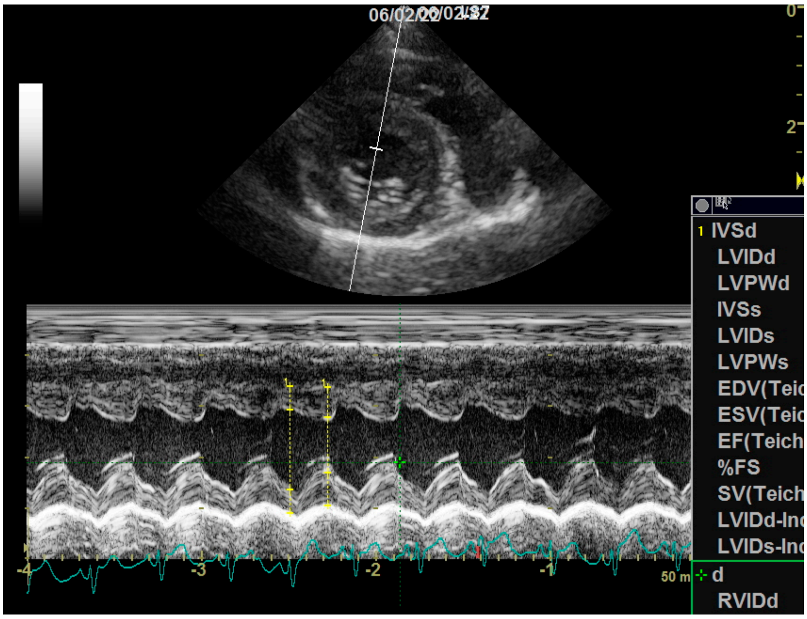

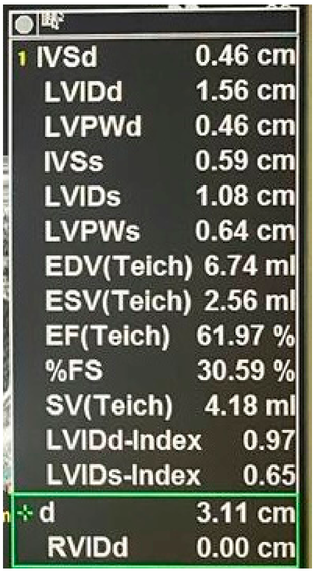

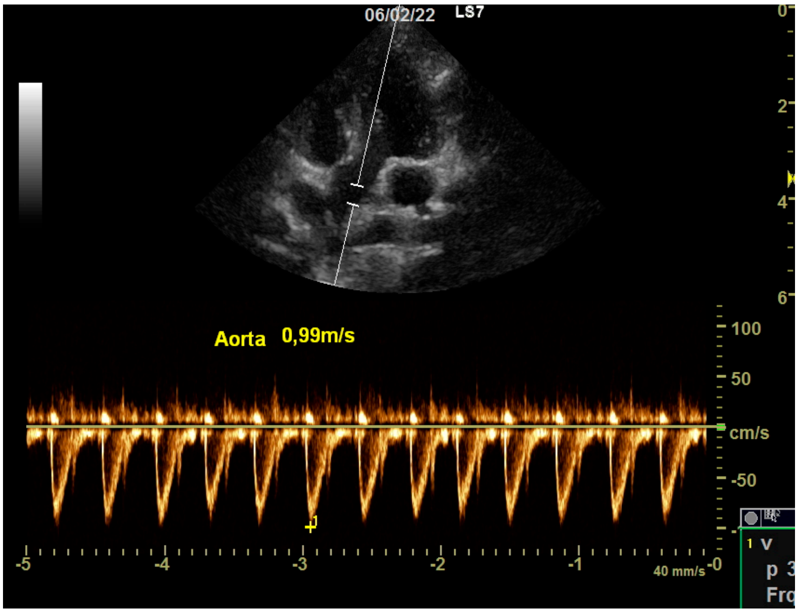
06/02/22
LS7
Aorta 0,99m/s
100
50
cm/s
-50
40 mm/s

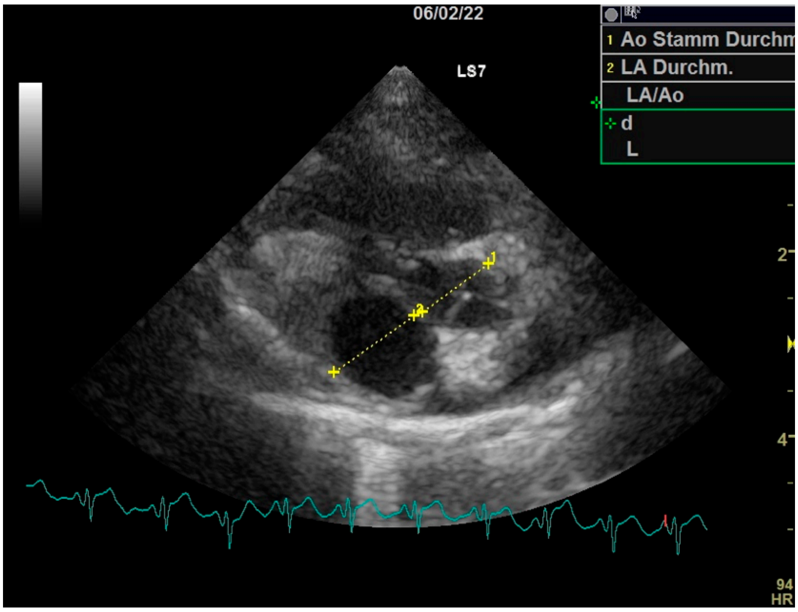
06/02/22
LS7
1 Ao Stamm Durchm
2 LA Durchm.
LA/Ao
d
L
94
HR

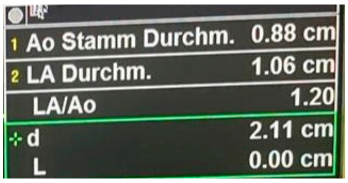
1 Ao Stamm Durchm. 0.88 cm
2 LA Durchm. 1.06 cm
LA/Ao 1.20
d 2.11 cm
L 0.00 cm

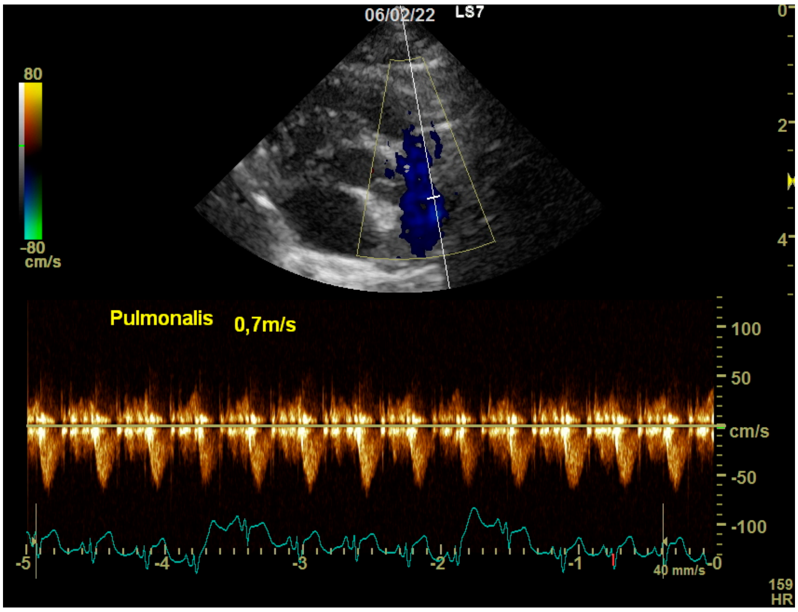
06/02/22
LS7
80
-80
cm/s
Pulmonalis 0,7m/s
100
50
cm/s
-50
-100
40 mm/s
159
HR
